# IMPACTS OF SOCIAL RELATIONSHIPS ON CO-OCCURRING SYMPTOMS AND FUNCTIONING IN OLDER ADULTS WITH COGNITIVE IMPAIRMENT

**DOI:** 10.1093/geroni/igac059.2059

**Published:** 2022-12-20

**Authors:** Bada Kang, Sijia Wei, Eleanor McConnell, Kirsten Corazzini

**Affiliations:** Yonsei University, Seoul, Seoul-t'ukpyolsi, Republic of Korea; Duke University, Durham, North Carolina, United States; Duke University, Durham, North Carolina, United States; University of New Hampshire, Durham, New Hampshire, United States

## Abstract

Social relationships are crucial for well-being of older adults with cognitive impairment (CI), however, evidence is lacking on how social relationships may influence symptom experience and functioning among those living with CI. This study aimed to identify subgroups of older adults with CI with distinct symptom and functioning profiles and to examine the association between latent class membership and social relationships. The sample included 927 older adults who were screened as having moderate or severe CI from wave 2 of the National Social Life, Health and Aging Project. Symptom (i.e., pain, fatigue, sleep disturbance, depression, anxiety, and stress) and functioning (i.e., instrumental activities of daily living, activities of daily living, and urinary incontinence) variables were used to identify subgroups. Latent profile analyses identified five distinct groups: most (51.7%) belonged to the low-symptom-high-functioning; few (7%) belonged to the high-symptom-low-functioning; while two groups had average symptom burden, one group (25%) had frequent urinary incontinence and normal daily functioning (poor-urinary-functioning), and the other group (5%) had normal urinary functioning and the worst daily functioning (worst-daily-functioning); interestingly, 13% belonged to high-symptom-normal-functioning group. Multinomial logistic regression modeling showed that, among social networks, support, strain, and engagement, members in groups with worse symptom burden and daily functioning were significantly more likely to have social strain, after adjusting for covariates (p-values < 0.01). Only severe CI was associated with worst-daily-functioning (OR = 3.24, p-value = 0.002). Interventions that ameliorate social strain may benefit symptom management and promote independent daily living among older adults with CI.

